# Differentiation of Fresh and Processed Fruit Juices Using Volatile Composition

**DOI:** 10.3390/molecules24050974

**Published:** 2019-03-10

**Authors:** Rosa Perestrelo, Catarina Silva, Pedro Silva, Sonia Medina, José S. Câmara

**Affiliations:** 1 CQM–Centro de Química da Madeira, Universidade da Madeira, Campus da Penteada, 9020-105 Funchal, Portugal; cgsluis@uma.pt (C.S.); pedro_dasilva@hotmail.com (P.S.); sonia.escudero@staff.uma.pt (S.M.); 2 Departamento de Química, Faculdade de Ciências Exatas e Engenharia, Universidade da Madeira; Campus da Penteada, 9020-105 Funchal, Portugal

**Keywords:** volatile pattern, fresh juice, processed juice, HS-SPME, GC-MS

## Abstract

In the current study, a comprehensive approach based on headspace solid-phase microextraction (HS-SPME), combined with gas chromatography-quadrupole mass spectrometry (GC-qMS), was used to establish the volatile signature of fresh and processed fruit juices, obtained from the same batch of grapes, red fruits, orange, pear, and apple. This is a powerful tool for evaluating the impact of the production process on the volatomic pattern of fruit juice. A total of 169 volatile organic compounds (VOCs) belonging to different chemical groups were identified. Esters, carbonyl compounds, terpenoids, and alcohols are the major chemical groups in the investigated fruit juices. However, their contribution to the total volatile profile varied. Special attention should be paid to processed fruit juices to avoid the possible deleterious effects associated with the formation of furanic compounds (e.g., heat treatment), since their furanic content was significantly higher in comparison to that of fresh fruit juices. The knowledge obtained in the current study will allow for the introduction of modifications to the process involved in processing juice, which will improve the organoleptic characteristics of processed juices, contributing to a better acceptance by consumers. Furthermore, more assays should be performed to assess the effect of harvests, geography, and agronomy on the volatile profile of juices.

## 1. Introduction

A balanced diet that includes fruit benefits health. Fruit consumption may contribute to interference with cancer mechanisms, assist in ameliorating mutagenic, inflammatory, and neurodegenerative mechanisms, as well as contribute to some antimicrobial effects [1,2]. It represents a rich source of vitamins (mainly vitamin C), pectin, fibers, organic acids, and secondary metabolites originating predominantly from plant metabolism, including volatile organic compounds (VOCs), phenolic compounds, etc. [3,4]. Furthermore, most of the fruits are used in many foodstuffs, such as jams, liquors, wine, dairy products, and breakfast cereals [3].

The volatile profile of fruits is responsible for their aroma and is constituted by a complex mixture of hundreds of VOCs belonging to different chemical groups. These VOCs are dominated by four biosynthetic classes: terpenoids, fatty acid derivatives, compounds with aromatic rings (derived from l-phenylalanine), and volatiles derived from amino acid metabolism (methyl-branched alcohols, ketones, esters, sulfur containing and aromatic compounds) [5,6]. More than 300 VOCs have been identified in the aroma profile of apples [5], esters being the most dominant chemical group detected in different apple cultivars. From this chemical group, 2-methyl butyl acetate, hexyl acetate, butyl acetate, 2-methyl butanoate, ethyl butanoate, and ethyl methyl propanoate were the most important esters contributing to the characteristic apple aroma [6,7,8,9]. In pear fruit, esters were also the most significant contributors to aroma [10], being methyl and hexyl esters of decadienoate, the character imparting compounds of the European pear [11]. Other esters, such as hexyl acetate, 2-methylpropyl acetate, butyl acetate, and pentyl acetate, as well as butyl butanoate, ethyl hexanoate possessed strong pear-like odors [12]. In addition, hexanal, 2-methylpropyl acetate, ethyl acetate, hexyl acetate, 3-methylbutyl-2-methyl butanoate, ethyl butanoate, and butanol were identified as impact volatiles in Conference pears (*Pyrus communis* L.) [13]. Orange is a large cultivated fruit and studied worldwide. A total of 58 VOCs, including esters, terpenoids, carbonyl compounds, alcohols, and acids were identified and quantified in Dortyol yerli orange juice. Terpenoids were the most representative chemical group, limonene being the predominant VOC, followed by valencene, linalool, terpinen-4-ol, and α-terpineol [14]. On the other hand, grapes (*Vitis vinifera* L.) volatiles include a great number of VOCs, namely monoterpenoids, sesquiterpenoids, carbonyl compounds, alcohols, C_13_ norisoprenoids, and acids [15]. Linalool, geraniol, citronellol, nerol, dienol I and II were the most abundant terpenoids in Muscat grape [16]. Other monoterpenes potentially contributing to Muscat aroma were rose oxide, citral, geraniol, nerol, and citronellol [17]. The evolution of terpenic compounds was evaluated during ripening of four *V. vinifera* L. grape varieties, Bual, Malvasia, Sercial (white grapes), and Tinta Negra (red grapes). Linalool, citronellol, geraniol, α-ylangene, bicyclogermacrene, β-ciclocitral, β-damascenone are common to all grape varieties studied and were detected in all ripening stages, whereas others were found only in some ripening stages and in some varieties (e.g., β-ocimene isomer, β-gurjunene, γ-elemene). In addition, for all *V. vinifera* L. grapes studied, the maximum potential of mono-, sesquiterpenic, and norisoprenoid compounds was achieved at maturity date, which was established based on the maximum sugar content and minimum titratable acidity [18]. Even at low levels, these VOCs may have a significant impact on the overall flavor of fruits and their corresponding juices due to their lower odor threshold (OT). The levels of these VOCs are frequently low (typically µg/L) and are influenced by a number of agronomic (e.g., variety, climatological conditions, ripening stage) [19] and technological factors (e.g., harvest, post-harvest treatments, storage and processing conditions) [20]. During fruit juice processing, the volatile signature is affected by the addition of additives, preservatives, and chemical or processing treatments that change the volatile profile of fresh juices [21]. 

Temperatures between 85 and 95 °C are frequently used to extend the shelf life of fruit juices However, it has been documented that the thermal process may activate the dormant ascospores of molds, which subsequently cause deterioration, hence resulting in economic loss [22]. Heat treatment with high temperature and long processing time is not recommended, due to quality reasons and consumer demands for fresh-like fruits. To overcome this problem on fruit juice preservation, non-thermal methods, such as high-pressure processing (HPP), supercritical carbon dioxide technology (SCCD), and power ultrasound in combination with mild heat have been proposed. Yi et al. [23] evaluated the impact of HPP (600 MPa, 3 min) on apple (Pink Lady, Granny Smith, and Jonagold) juice quality changes and compared it to the thermal processing. The overall quality of HPP treated apple juices was much more comparable to that of the fresh juice, in particular HPP results in lower amounts of thermally induced compounds that are related to cooked notes of pasteurized apple juices. Kebede et al. [24] investigated the volatile changes during the shelf life of pulsed electric fields (PEF) (15.5 kV/cm and specific energy of 158 kJ/L), HPP (600 MPa for 3 min), and thermally (72 °C for 15 s) pasteurized Jazz apple juices, up to five weeks. The results showed that after pasteurization, PEF processing resulted in a better retention of odor active VOCs, such as 2-hexenal isomer and hexyl acetate, whereas thermal processing lowered their amount. Gao et al. [25] evaluated the effects of HPP (550 MPa/10 min) and high-temperature short time (HTST; 110 °C/8.6 s) on microorganisms, ascorbic acid, total phenols, antioxidant capacity, color, enzyme activity, and rheological behavior in red grapefruit juice during 30 days of storage at 4 °C. Evelyn et al. [26] showed that HPP pretreatment enhanced the thermosonication inactivation of *Alicyclobacillus acidoterrestris* spores in orange juice, whereas Mastello et al. [27] evaluated the impact of HPP processing on the volatile profile and sensory acceptance of Pêra-Rio orange juice. Moreover, several studies have demonstrated the efficiency of SCCD processing in the preservation of juices such as orange, melon, kiwi, pear, and strawberry. The findings confirm the SCCD processing as a potential promising technology to the conventional thermal treatment [28,29,30]. The assessment of the volatile signature of fruit juice is of great importance, since it enables researchers to help producers improve its quality, develop new products for the market, contribute to its economic valorization [27], as well as to assess the geographical origin, authenticity, and typicity of food matrices [31]. 

Several extractions and enrichment techniques, namely, liquid–liquid extraction (LLE) [32], simultaneous distillation extraction (SDE) [33], solid-phase trapping solvent extraction (SPTE) [33], solid-phase extraction (SPE) [34], pressurized-fluid extraction (PFE) [35], solid-phase microextraction (SPME) [3,33], and stir bar sorptive extraction (SBSE) [36] have been applied to establish the volatile signature of food matrices. Nevertheless, microextraction techniques (METs) (e.g., SPME, SBSE) compared to conventional extraction techniques (e.g., LLE, SDE, SPE) offer several advantages, such as a reduction of the amount of organic solvent, the possibility of automation, high sensitivity and concentration factors [37]. These METs, combined with analytical measurements, can be used to assess the sensory properties, chemical composition, authenticity, and typicality of juices. The establishment of the volatile signature, analysis of the nutritional composition, phenolic compounds, carotenoids, and some vitamins [3,38], in addition to sensory analysis using consumers and/or a trained taste panel, or even using an instrumental taste sensing system, like the electronic tongue [39], are the most widely used techniques to assess the quality of fruit juices [40]. 

The main goal of this study was to establish and compare the volatile signature of fresh and processed fruit juices, obtained from the same batch of fruits, by means of headspace solid-phase microextraction (HS-SPME), combined with gas chromatography-quadrupole mass spectrometry (GC-qMS). In addition, the impact of processing on the volatile signature of juices from grapes (*Vitis vinifera* L.), wild red fruits (*Fragaria* x *ananassa Duch*., *Vaccinium corymbosum* L., *Rubus idaeus* L.), orange (*Citrus sinensis* L.), pear (*Pyrus communis* L.), and apple (*Malus domestica Borkh*.), as well as the corresponding processed juices (from the same batch), was investigated. These comprehensive data will allow the evaluation of the impact of processing on the volatile signature of fruit juices from the same batch. As far as we know, this is the first time that different fruit juices corresponding to different fruit species were analyzed simultaneously using the same analytical technique, therefore enabling us to describe both the volatile profile in the juice and the variability in the volatile profile between fresh and processed fruit juices. The obtained results may help to enlarge the database on the volatile and aroma composition of fresh and processed fruit juices, as well as provide a useful platform to improve the organoleptic characteristics of the processed juices and consequently contribute to their economic valorization and improve their acceptance by consumers. 

## 2. Results and Discussion

### 2.1. Volatomic Signature of Fresh and Processed Fruit Juices

**Figure 1:**
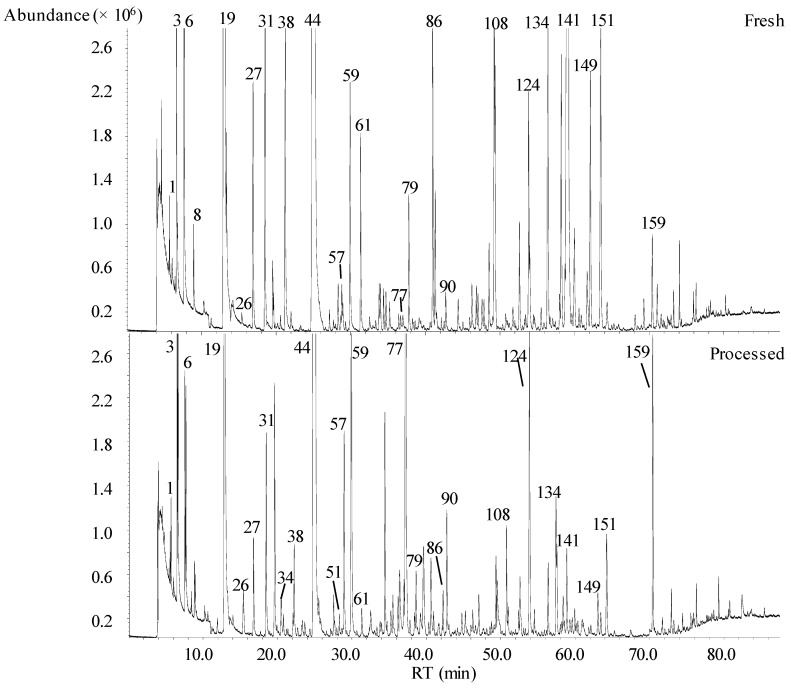
Total ion chromatograms obtained by the headspace solid-phase microextraction (HS-SPME)_DVB/CAR/PDMS_/gas chromatography-quadrupole mass spectrometry (GC-qMS) analysis of fresh and processed pear juice (for the identification of peak numbers, see Table 1).

A comprehensive untargeted analysis of the volatile signature of fresh and processed fruit juices, obtained from the same batch of fruits, was performed using HS-SPME/GC-qMS. The data were processed using software (NIST, 2005; Mass Spectral Search Program V.2.0d, Agilent, Washington, USA), that provides quality matching using advanced spectral matching algorithms background subtraction and kovats index (KI) comparison. Figure 1 shows a typical total ion chromatogram (TIC) of fresh (top) and processed (bottom) pear juices obtained by HS-SPME/GC-qMS. Great differences among the patterns of the VOCs from fresh and processed pear juices were observed. On average, 125 VOCs were identified in the headspace of the investigated processed fruit juices (108 in grape, 117 in red fruits, 97 in orange, 123 in pear, and 124 in apple juice), whereas the volatile signature of fresh juices was less complex, with 111 VOCs identified (84 in grape juice, 111 in red fruits, 98 in orange, 108 in pear, and 109 in apple juices) (Figure S1, Supplementary Materials). The identified VOCs belonged to distinct chemical groups, including terpenoids (57), esters (56), carbonyl compounds (33), alcohols (16), acids (3), volatile phenols (2), and furanic compounds (2) (Table 1). Moreover, some differences were also observed in both the qualitative and semi-quantitative (GC peak area) expressions. Table 1 shows the KI determined with a BP20 column, as well as the KI reported in the scientific literature with equivalent columns, was used to confirm the identity of VOCs. Thirty-six of the identified VOCs were common to all the fresh and processed juices investigated (Table S1, Supplementary Materials). On the other hand, some VOCs were detected in specific juices, such as 3-methylbutanal in grape juice, hexyl butanoate and rose oxide isomer in red fruit juices, p-cymene, safranal, and carvone in orange juice, ethyl pentanoate and geranyl valerate in apple juice, and geranial in pear juice.

**Figure 2:**
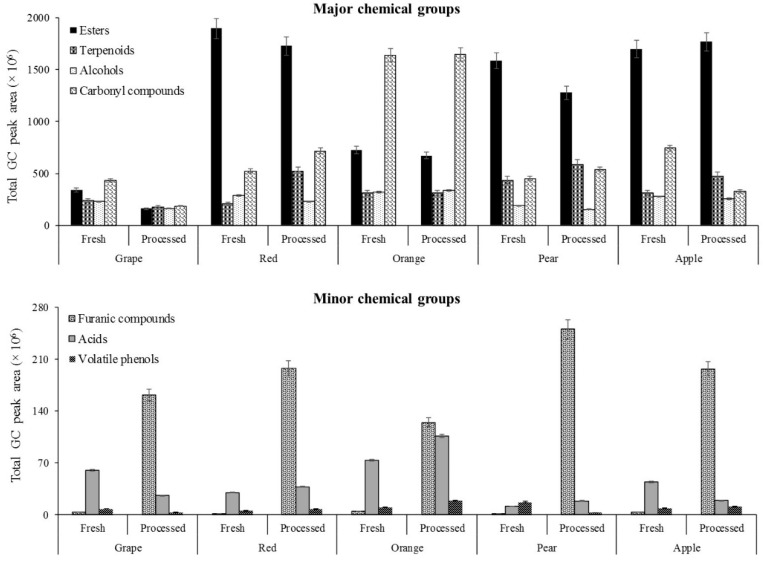
Total GC peak area of chemical groups identified in fresh and processed fruit juices.

The distribution of the chemical groups, according to the production process of juices, is shown in Figure 2. Esters, carbonyl compounds, terpenoids, and alcohols are the predominant chemical groups detected in fruit juices. However, significant differences were observed among these chemical groups, according to the production process and fruit variety. The esters were the predominant chemical group in all of the investigated fruit juices (55.2%, on average), except for orange and grape juices, which account for 23.6 and 26.0% of the total volatile profile of the fresh juices, and 20.9 and 18.5% of that of processed juices, respectively. However, the GC total peak areas of esters were, on average, 1.1 and 1.4 times higher in fresh apple juices than the corresponding processed juices. Hexyl acetate was the most abundant ester identified in red fruits, pear, and apple juices, irrespective of the type of juice (Table 1 and Table S2).

In orange and grape juices, terpenoids was the most predominant chemical group, accounting for 53.0 and 32.9% of the total volatile composition of fresh juices, and 51.2 and 21.4% of that of processed juices, respectively. While the number of identified terpenoids in orange, pear, and apple juices was similar, its GC peak area in fresh and processed orange juices was three and four times higher than observed in fresh and processed juices from pear and apple, respectively. This trend is explained by the high GC peak areas of limonene, which were followed by those of germacrene D, geranyl acetone isomer and linalool, observed in orange juices (Table 1 and Table S2). On average, limonene represents 11.6 and 6.3% of the total volatile profile of fresh and processed juices, respectively. 

On the other hand, the number of terpenoids identified in orange juice, independently of the type of juice, was two times higher than that in grape juice. The contribution of this chemical group to the total volatile profile of grape juice was two times higher than its contribution to that of red fruits, apple, and pear juices, irrespective of the type of juice (Table 1). Dihydrolinalool, followed by linalool and α-terpineol, were the predominant terpenoids identified in grape juices (Table S2).

In the studied fresh juices, the contribution of alcohols to the total volatile profile ranged from 10.1 (orange) to 18.1% (grape), whereas in processed juices its contribution ranged from 9.7 (orange) to 20.7% (pear). The ethanol content was higher in the fresh juices from grape, orange, and pear than in the corresponding processed juices, whereas in the red fruit juices and apple juices, the GC peak area of ethanol was higher in the processed juices than in the fresh juices. 1-Propanol was the second most dominant alcohol in grapes and red fruit juices, whereas 3-methyl-1-butanol was the most dominant alcohol in orange and apple juices. 

Regarding furanic compounds, a significant increase in terms of the total GC peak area (Figure S3) and % RPA (Table 1) was observed for all investigated processed juices, unlike the fresh juices. The contribution of this chemical group to the total volatile profile of processed juices ranged from 3.9 (orange) to 18.3% (grape), whereas in fresh juices, its content was significantly low (*p* < 0.05), ranging from 0.03 (red fruits) to 0.3% (grapes).

### 2.2. Multivariate Data Analysis

**Figure 3:**
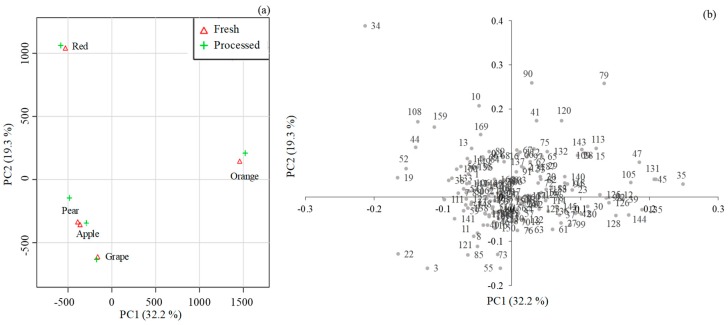
Principal component analysis (PCA) of the volatile signature of fresh and processed fruit juices. (**a**) PC1 × PC2 score scatter plot and (**b**) loading weight plot (attribution of the peak number is shown in Table S1).

The data matrix (variables versus fresh and processed fruit juices) was normalized using cubic root and data scaling by the mean-center and was submitted to principal component analysis (PCA) to explore the main sources of similarity and variability as well as to characterize and differentiate the target types of fruit juices, fresh versus processed, based on the volatomic signature (Figure 3). 

For all of the analyzed fruit juices, the PCA score scatter plot of the two first principal components (PC1 (32.2%) and PC2 (19.3%)), which explain 51.5% of the total variability of the GC-qMS dataset, is shown in Figure 3a, and the corresponding loading weight plot, establishing the magnitude of each VOC (variable), is illustrated in Figure 3b. Analysis of the loading plot revealed the VOCs responsible for the discrimination of fruit juices based on fruit species/type. Fresh and processed red fruit juices, projected in PC1 negative and PC2 positive, were mainly characterized by methyl hexanoate (34), whereas apple, pear, and grape juices (PC1 and PC2 negative) were mainly characterized by ethyl acetate (3) and 3-methylbutanol acetate (22). Finally, orange juices projected in PC1 and PC2 positive were mainly associated with limonene (35).

**Figure 4:**
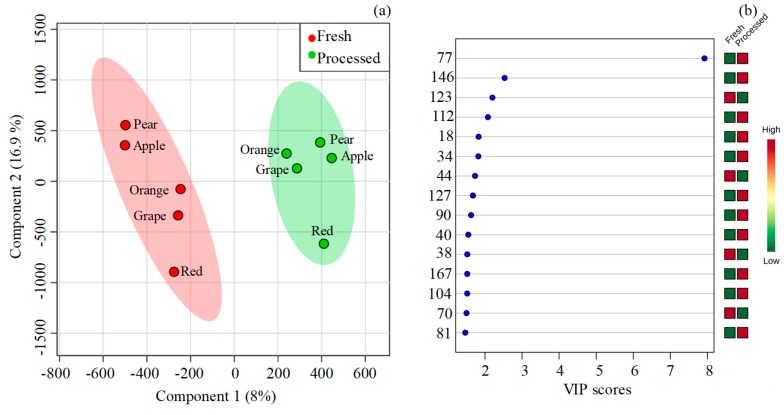
Partial least squares-discriminant analysis (PLS-DA) of the volatile signature of fresh and processed fruit juices. (**a**) Score scatter plot, and (**b**) Variable importance in projection (VIP) scores (attribution of the peak number is shown in Table 1).

Additionally, the partial least squares-discriminant analysis (PLS-DA) (Figure 4a,b) was used as a supervised cluster, and 30 differently expressed VOCs were found with variable importance in projection (VIP) scores higher than 1: 2-furfural (77), ethyl dodecanoate (146), valencene (123), ethyl 3-hydrohexanoate (112), ethyl 3-methylbutanoate (18), methyl hexanoate (34), hexyl acetate (44), 2,4-decadienal (127), linalool (90), and propyl butanoate (40) were the most significant. Figure S3 shows the resulting dendrogram associated with the heat map constructed using Pearson´s correlation, providing an intuitive visualization of the dataset, which is often applied to identify samples or features that are unusually high or low. Five major clusters were responsible for grouping the juice samples by fruit variety (horizontal axis), and not by the type of juice (fresh or processed). An analogous color tone in the heat map indicated the area, a group of samples, considering that the concentration of the analyzed VOCs was similar. 

**Figure 5:**
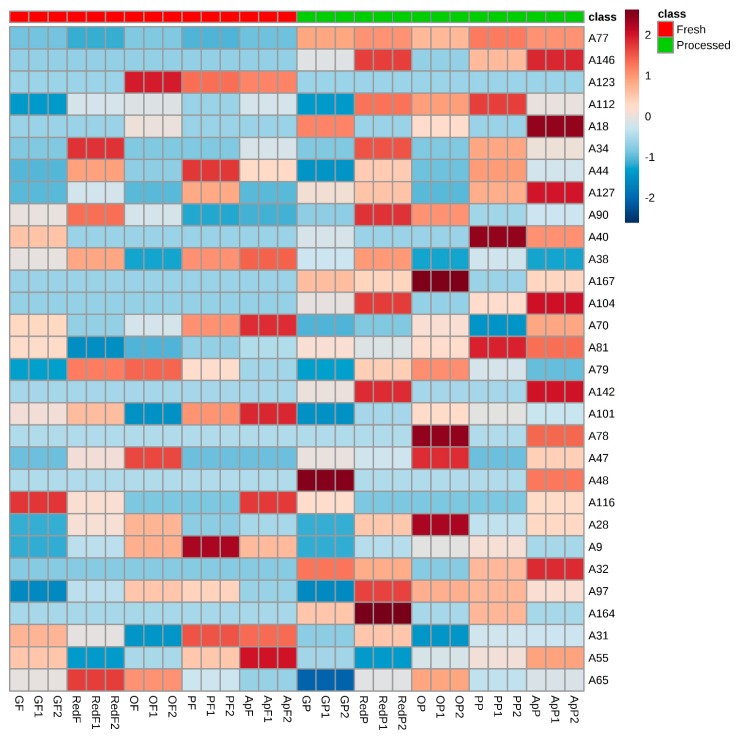
Hierarchical cluster analysis (HCA). The heat maps of the 30 VOCs (VIP values > 1), identified in fresh and processed fruit juices were generated by an average algorithm and Pearson’s distance analysis (attribution of the peak number is shown in Table 1). Abbreviation: OP—orange juice processed, OF—orange juice fresh, GP—grape juice processed, GF—grape juice fresh, PP—pear juice processed, PF—pear juice fresh, ApP—apple juice processed, ApF—apple juice fresh.

Figure 5 shows the heat map constructed using Pearson´s correlation for the VOCs with VIP scores higher than 1. It is possible to observe that 2-furfural (77), ethyl dodecanoate (146), ethyl 3-hydrohexanoate (112), ethyl 3-methylbutanoate (18), 4-ethy phenol (167), butyl octanoate (104), 2-heptanone (32), and γ-decalactone (164) had a higher chromatographic area in processed juice than in fresh fruit juice, whereas valencene (123) had a higher chromatographic area in fresh than in processed fruit juice.

## 3. Discussion

In sum, fruit aroma is an important indicator, which may reflect the quality of future fruit juice. The contribution of each VOC to the specific aroma signature of each fruit species/types depends on the activity and substrate specificity of the relevant enzymes in the biosynthetic pathway, the substrate availability, the OT above which VOC can be detected by smell, and the presence of the other VOCs [5]. The esters represented more than 45.15% of the total volatile profile of apple, pear, and red fruit juices, which is in accordance with previous studies that report esters as the most abundant chemical group in pear and apple fruit juices [6,7,11,13]. Regarding this chemical group, hexyl acetate, a dominant esters in pear juice, has been reported as possessing a strong pear-like odor [12]. Moreover, ethyl octanoate contributes to sweet and fruit odors in pears, while hexanal, ethyl acetate, hexyl acetate, ethyl butanoate, and butanol are identified as impact volatiles in Conference pears [13]. On the other hand, terpenoids, one of the most dominant chemical groups of VOCs found in orange and grape fruit juices, have shown beneficial functions as well as nutraceutical activities. Recently, some studies in the field of cancer research and food nutrition sciences have been performed on these VOCs due to their potential anticancer properties [44]. Among them, limonene (a dominant VOC in orange juice) has been reported to be a bioactive food component from citrus fruits, with a potential role in breast cancer prevention and treatment [45]. In addition, limonene reduces the oxidative stress in Streptozotocin-induced diabetic rats by decreasing lipid peroxidation and sparing the activities of antioxidant enzymes [46]. Linalool (a dominant VOC in red fruit juice) and terpineol (a dominant in grape fruit juice) are responsible for the fruity and floral notes of food matrices. Moreover, linalool and its degradation product, α-terpineol, have been reported as potent antimicrobial agents against periodontopathic and cariogenic bacteria [47]. Anti-inflammatory effects were also associated with these terpenes [48]. α-Farnesene, a specific terpenoid, with green and herbaceous aroma descriptors, has been reported as the most abundant terpene in fresh apple juice [49], which is in agreement with the results of our study, since we detected GC peak areas (×10^6^) in fresh and processed apple juices of 419.32 and 8.16, respectively. Moreover, according to previous studies, the aroma of squeezed orange juice is primarily associated to esters (e.g., ethyl butanoate, ethyl 2-methylpropanoate), aldehydes (e.g., acetaldehyde, hexanal, octanal), in addition to a smaller number of terpenoids (e.g., myrcene, α-pinene, potentially limonene, linalool, geranial). All these VOCs are responsible for the citrus, green, sweet fruit, and floral notes from fresh orange juice [50,51,52]. 

There are differences between the VOCs contained in fresh and processed fruit juices, with processed juices having the highest levels of VOCs. Indeed, a recent study of fresh and commercial pomegranate juice indicated that there were marked differences between endogenous VOCs extracted in the laboratory versus what was often found in commercial products [53]. This fact may be due to several characteristics of fruits (e.g., geographical and botanical origin, varieties, ripening stage, farming practices, and post-harvest handling), the process applied in juice extraction and its production (e.g., enzymes, heat, and filtration), as well as storage conditions [5]. A recent assay reported that the volatile loss generally continued through pasteurized storage, and the lowest volatile levels were recovered after one, two, and four months of storage [54]. Another study highlighted that changes in volatile chemicals could provide useful information about juice processing for the selection of the optimal harvest time and for setting a precise blending strategy [55]. Our study showed that the volatile profile of processed juices is more complex than that of fresh juices. This result is in accordance with a previous work carried out by Schmutzer and colleagues [49], in which fresh apple juice was compared with commercial apple juice. These authors highlighted that the differentiation criteria between fresh and commercial juices based on individual compounds implied a difficult characterization process, which is not helpful in establishing a quality index. Consequently, the most appropriate and useful method is to compare groups of VOCs (in our study: esters, alcohols, carbonyl compounds, terpenoids, volatile phenols, acids, and furanic compounds). In general, fresh juices were mainly characterized by the presence of esters and terpenoids, while furanic compounds (Maillard products) were important contributors to the profile of processed juices. Our findings are in accordance with a previous report concerning pomegranate juices [56]. Moreover, storage time and temperature can also change juice aroma profiles due to Maillard, strecker, and acid catalyzed hydration reactions. The formation of methional from methionine is probably the most significant example of Strecker degradation in orange juice [51]. Methional concentration in processed orange juice is higher than fresh juice (Table 1). Moreover, esters as stated above represent an important contribution to fruit juice odor, and the level of total esters in aqueous essence has been used as a quality index [51]. Nevertheless, their concentration in processed juice was lower than fresh juice, since during processing many esters rapidly hydrolyzed by hydrolase enzymes.

The presence of ethanol in the investigated fruit juices is indicative of the fermentation process caused by microorganisms as a consequence of inefficient sterilization of raw materials or later contamination. A previous study reported that high ethanol contents were observed mainly in juices without preservatives, particularly freshly-squeezed juices. Octanal and decanal, the most dominant compounds, of the carbonyl group found, in orange juice unlike other juices, have been considered to be important contributors to the orange flavor and standard of orange peel oil [57]. An important point to highlight is that the production of excessive volatile phenols is regarded as spoilage. In our study, 4-ethyl phenol, in particular, was only detected in processed juices, suggesting a possible microbial contamination (e.g., *Brettanomyces* sp). It has been reported [58] that this phenol may provide woody, smoky, leather, and animal off-odors. In the same sense, Daud and colleagues [59] reported that another family of compounds, which may contribute to undesirable smells or odors, is acidic compounds such as octanoic and hexanoic acids.

The data obtained in the current study represent an added value to the existing knowledge, as well as provide a useful platform to improve the organoleptic characteristics of the processed juices, and consequently contribute to their economic valorization and improve their acceptance by consumers.

## 4. Materials and Methods

### 4.1. Material and Chemicals

All chemicals were of analytical quality. Sodium chloride (NaCl, 99.5%) was supplied from Panreac (Spain, Barcelona) and the VOC standards used for identification were purchased from Sigma–Aldrich (Madrid, Spain) and Acros Organics (Geel, Belgium), with a purity ≥ 98%. Helium of purity 5.0 (Air Liquide, Lisbon, Portugal) was utilized as the GC carrier gas. The glass vials, SPME fibers and SPME holder for manual sampling were purchased from Supelco (Bellefonte, PA, USA). The KI was calculated through the injection of a series of C_8_ to C_20_ straight-chain n-alkanes (concentration of 40 mg/L in *n*-hexane), supplied by Fluka (Buchs, Switzerland).

### 4.2. Fruit Juice Samples

Healthy mature-state and undamaged fruit samples from fresh grapes (*Vitis vinifera* L.), wild red fruits (*Fragaria* x *ananassa Duch*. *Vaccinium corymbosum* L., *Rubus idaeus* L.), orange (*Citrus sinensis* L.), pear (*Pyrus communis* L.), and apple (*Malus domestica Borkh*.) were collected at maturity state based on the sugar/acid ratio, color, and pH from producers in the southwest region of Madeira Island, Portugal during the 2016 harvest and were immediately taken to the process line at 4 °C. For each sampling, fruits were picked randomly throughout the experimental vineyard, taking into consideration the balance between shadow and sun exposure in the different vineyard locations, following a z-shaped pattern to avoid edge and center effects. In the process line, the fresh fruits were screened, inspected, washed, mechanically crushed, and submitted to an enzymatic treatment using pectinase enzyme (concentration of enzyme: 5 mg/100 g; incubation time: 2 h; and temperature: 35 °C). Operations such as washing, sorting and inspecting require attention to mass and heat transfer. Cooling depends upon heat transfer from fruit to air (possibly water). Cleaning sometimes involved the physical removal of surface debris by brushes which reduced the number of microbes. Inspecting sometimes involved the removal of raw materials which did not meet the standards of the quality of analysis. The enzymatic activity was monitored by adding five milliliters of juice to 15 mL of HCl-acidified ethyl alcohol, observing the mixture for 5 min for gel formation. No gel formation meant that the depectinization was complete. After this, the juice was extracted at room temperature (25 °C), using the equipment appropriate for each fruit variety, and then filtrated (XP-201 polyvinylidene fluoride membrane) under vacuum. At this stage, fresh fruit juices were taken, in 250-mL amber glass vials and immediately transported to the laboratory at 4 °C, aliquoted in 20-mL amber glass vials and frozen at −80 °C until analysis. The remaining fruit juice was submitted to a pasteurization process at 80–95 °C for 1 to 10 min in intermediate tanks to eliminate bacteria without changing the fresh flavor of the fruit juice. After this process, the fruit juice was submitted to a cooling batch (4 to 5 °C) and sterile storage. At this stage, the processed fruit juice was taken, transported to the laboratory, aliquoted, and frozen at −80 °C until analysis. All analyses were performed in triplicate.

### 4.3. Headspace Solid-Phase Microextraction

The HS-SPME experimental parameters used in this work were previously optimized in several works, developed in our group [15,19,41,60]. Briefly, aliquots of 5 mL of juice samples were placed into a 10-mL glass vial. After the addition of 10% (*w*/*v*) of NaCl and stirring (0.5 × 0.1 mm bar) at 400 rpm, the vial was capped with a PTFE (polytetrafluoroethylene) septum and an aluminum cap (Chromacol, Hertfordshire, UK). The vial was placed in a thermostated bath, adjusted at 40.0 ± 0.1 °C for 5 min, and then the 50/30 μm divinylbenzene/carboxen/polydimethylsiloxane (DVB/CAR/PDMS) fiber was inserted into the headspace for 45 min. Each sample was analyzed in triplicate. Blanks, corresponding to the analysis of the coating fiber and not submitted to any extraction procedure, were run between sets of three analyses.

### 4.4. Gas Chromatography-Mass Spectrometry Conditions

The gas chromatography mass spectrometry conditions were previously established [15,41]. The fiber of the SPME containing the VOCs was manually introduced into the GC injection port at 250 °C (equipped with a glass liner, 0.75 mm I.D.) for 5 min for desorption. The desorbed VOCs were analyzed in an Agilent Technologies 6890N Network gas chromatography equipped with a BP-20 fused silica capillary column (30 m × 0.25 mm I.D. × 0.25-μm film thickness) supplied by SGE (Darmstadt, Germany), connected to an Agilent 5973N quadrupole mass selective detector. Helium (Air Liquid, Lisbon, Portugal) was used as the carrier gas at a flow rate of 1.0 mL/min (column-head pressure: 12 psi). The injections were performed in the splitless mode (5 min). The GC oven temperature was programmed as follows: 45 °C (1 min), then increased by 2 °C/min to 100 °C (3 min), 5 °C/min to 130 °C (5 min), and finally 20 °C/min to 220 °C (2 min). For the MS system, the temperatures of the transfer line, quadrupole, and ionization source were 250, 150, and 230 °C, respectively. The electron impact mass spectra were recorded at 70 eV, and the ionization current was about 30 μA. The acquisitions were performed in full scan mode (30–300 *m*/*z*). The identification of the VOCs was achieved by (i) comparing the GC retention times and mass spectra, with those, when available, of the standard compounds; (ii) comparing all mass spectra with the data system library (NIST, 2005 software, Mass Spectral Search Program V.2.0d, Agilent, Washington, USA); and (iii) determining the kovat index (KI) values, according to the van den Dool and Kratz equation [61]. For the KI determination, a C_8_–C_20_ n-alkanes series was used, and the values were compared, when available, with values reported in the literature for similar columns [34,41,42,43,62].

### 4.5. Statistical Analysis

The obtained data were analyzed with Metaboanalyst 4.0 [63], which included data pre-processing to remove VOCs with missing values (MVs), MVs imputation of the resulting data (by Bayesian principal component analysis, PCA method), and normalization (data transformation by cubic root and data scaling by the mean-center). The normalized data were further subjected to one-way ANOVA, followed by Tukey’s test (*p* < 0.05), for post-hoc multiple comparisons of means and multivariate statistical analysis, namely, principal component analysis (PCA) and partial least squares-discriminant analysis (PLS-DA). This provided insights into the separations between the fresh and processed fruit juices and allowed VOCs that may have indicated differences among the samples sets to be identified. Finally, Pearson’s correlation was used to build the heat map of the fresh and processed fruit juices. 

## 5. Conclusions

The HS-SPME/GC–qMS has proven to be a suitable and solvent-free approach to the establishment of the volatile signature of fresh and processed juices from different fruits (pear, orange, red fruits, grape, and apple). This analytical technique, combined with multivariate analysis, may offer to fruit juice industries an alternative technique for routine analysis in the monitoring of processes that may affect aroma compounds in order to obtain fruit juices with high-quality flavors. For processed juices, special attention should be paid during the production process in order to avoid possible deleterious effects associated with the formation of furanic compounds, since a significant increase was observed from fresh to processed fruit juices of the same batch. Storage time and temperature can also change juice aroma profiles due to Maillard, Strecker, and acid catalyzed hydration reactions. In this sense, methional is probably the most significant example of Strecker degradation in orange juice, and it was possible to observe that its concentration in processed fruit juice was higher than in fresh fruit juice. On the other hand, esters contribute positively to fruit juice odor, with fruit and floral notes. However, their relative concentration in processed fruit juice was lower than fresh fruit juice, since during processing many esters rapidly hydrolyzed by hydrolase enzymes. Consequently, it was expected that their contribution to volatile profile on processed fruit juice was lower. These findings represent a suitable tool for providing data on fruit markers, which may be useful in defining the production procedures (e.g., enzyme, filtration, heat treatment, pasteurization, packing or storage), improving the organoleptic characteristics of the corresponding juices, and consequently contributing to the economic valorization and consumer acceptance of juices. Moreover, since the data obtained in the study correspond to one harvest, more studies need be conducted to investigate the typicality and reproducibility of the patterns of VOCs, across multiple harvests, and geographical and agronomic conditions.

## Figures and Tables

**Table 1:** GC peak area (×10^6^) of volatile organic compounds (VOCs) identified in fresh and processed fruit juices.

Peak n°	RT (min) ^1^	KI_Cal_^2^	KI_Lit_^3^	Chemical Groups	GC Peak Area (×10^6^) (RSD)																			
					Grape				Red Fruits				Orange				Pear				Apple			
					Fresh		Processed		Fresh		Processed		Fresh		Processed		Fresh		Processed		Fresh		Processed	
				**Esters**																				
1	5.67	897	864	Methyl acetate	11.18	(2)	2.17	(5)	29.89	(7)	16.76	(8)	1.23	(2)	1.98	(5)	23.94	(16)	21.71	(13)	1.98	(19)	2.58	(8)
3	6.59	925	907	Ethyl acetate ^4^	51.31	(3)	12.45	(5)	4.58	(6)	14.56	(15)	7.83	(11)	3.08	(15)	222.09	(13)	257.16	(10)	144.12	(8)	131.55	(16)
7	8.38	969	950	Ethyl propanoate ^4^	7.97	(6)	26.03	(5)	10.21	(4)	23.32	(7)	-		-		-		-		28.73	(6)	7.32	(12)
8	8.95	981	969	Propyl acetate ^4^	5.66	(6)	0.70	(9)	-		-		-		-		9.82	(5)	35.95	(1)	0.76	(18)	17.32	(1)
10	9.29	988	982	Methyl butanoate	-		-		94.87	(12)	46.71	(8)	1.27 ^a^	(3)	1.45 ^a^	(1)	-		-		6.14	(4)	1.51	(13)
11	10.17	992	1007	Methyl 2-methylbutanoate	12.72	(11)	1.48	(13)	1.06	(3)	-		-		-		2.15	(3)	4.52	(16)	10.32	(9)	12.34	(5)
13	10.53	1002	1015	Methyl 3-methylbutanoate	-		-		13.65	(3)	17.86	(6)	-		-		3.20	(1)	5.41	(9)	-		-	
15	11.33	1024	1028	Ethyl butanoate ^4^	17.95	(6)	6.42	(3)	30.48	(8)	18.24	(8)	64.68	(10)	62.50	(5)	-		-		10.08	(6)	1.94	(8)
16	12.08	1043	1050	Ethyl 2-methylbutanoate	-		-		1.72	(18)	42.60	(5)	2.35	(12)	1.94	(9)	-		-		21.85	(9)	2.19	(15)
18	12.75	1059	1060	Ethyl 3-methylbutanoate	-		10.39	(4)	-		-		1.25	(4)	2.16	(6)	-		-		-		40.51	(2)
19	13.02	1065	1075	Butyl acetate ^4^	2.18	(11)	2.34	(17)	67.76	(2)	56.12	(4)	-		-		21.32	(4)	16.93	(6)	132.24	(13)	221.32	(8)
22	15.40	1115	1117	3-Methylbutanol acetate ^4^	10.03	(13)	0.88	(11)	19.70	(7)	5.20	(15)	-		-		6.55	(12)	19.26	(14)	617.98	(12)	745.10	(8)
25	16.18	1130	1120	Ethyl pentanoate	-		-		-		-		-		-		-		-		6.03	(13)	11.65	(5)
26	16.51	1136	1120	Butyl propanoate	-		-		6.05	(14)	1.46	(7)	11.25	(3)	26.43	(3)	22.63	(1)	40.01	(9)	33.36	(18)	49.34	(4)
28	17.65	1156	1160	Propyl propanoate	-		-		5.51	(7)	10.41	(9)	14.16	(4)	66.78	(6)	0.65	(13)	1.97	(6)	1.18	(9)	7.71	(6)
30	17.88	1159	1152	Ethyl 2-butenoate	6.73	(10)	0.52	(13)	-		-		15.49	(14)	27.52	(2)	-		-		3.26	(3)	-	
31	18.52	1170	1147	Pentyl acetate	7.25	(8)	0.78	(11)	2.78	(8)	5.89	(3)	-		-		16.30	(9)	2.02	(9)	13.85	(14)	1.90	(6)
34	19.28	1182	1188	Methyl hexanoate	-		-		564.99	(7)	430.05	(7)	-		-		-		158.81	(3)	13.23	(8)	28.72	(9)
40	22.23	1229	1228	Propyl butanoate	4.59	(6)	0.74	(15)	-		-		-		-		-		48.35	(7)	-		10.56	(15)
41	22.36	1231	1220	Ethyl hexanoate ^4^	27.68	(11)	1.19	(5)	77.27	(13)	82.60	(15)	23.42	(7)	52.65	(15)	1.82	(16)	6.00	(9)	34.09	(7)	2.50	(1)
44	24.85	1269	1270	Hexyl acetate ^4^	31.63	(4)	12.66	(12)	356.35	(8)	233.56	(5)	53.36	(9)	39.23	(2)	676.33	(6)	368.37	(1)	193.62	(10)	109.58	(5)
49	26.85	1296	1292	Ethyl 3-hexenoate ^4^	-		-		3.06	(12)	1.28	(2)	-		-		2.09	(14)	4.47	(5)	1.01	(14)	-	
50	27.11	1300	1305	Ethyl 2-hexenoate	2.58	(9)	1.14	(12)	3.25	(5)	8.77	(4)	-		-		11.41	(5)	3.99	(8)	0.98	(5)	3.36	(8)
51	27.51	1306	1304	Ethyl 3-ethoxypropanoate	-		-		-		-		-		-		-		22.33	(5)	4.02	(15)	-	
52	27.76	1310	1328	3-Hexen-1-ol acetate isomer	4.27	(3)	3.36	(3)	36.28	(2)	142.79	(2)	-		-		5.91	(14)	9.81	(7)	78.43	(14)	56.48	(5)
54	28.31	1319	1327	2-Hexen-1-ol acetate isomer	-		0.25	(12)	5.57	(7)	4.12	(1)	-		-		27.12	(9)	11.09	(8)	3.32	(17)	2.88	(6)
56	29.10	1332	1332	Hexyl propanoate	-		0.76	(11)	26.11	(4)	7.36	(11)	-		-		6.93	(19)	3.66	(7)	4.42	(12)	4.52	(12)
57	29.37	1336	1305	Ethyl 2-hexenoate	3.12	(5)	8.58	(13)	0.39	(6)	2.53	(4)	2.01	(5)	5.47	(8)	1.52	(17)	2.24	(7)	6.87	(16)	3.75	(1)
61	31.36	1366	1359	Heptyl acetate	5.91	(12)	0.33	(9)	-		-		12.28	(8)	23.95	(7)	20.09	(14)	6.38	(4)	1.26	(7)	30.99	(3)
64	32.35	1380	1389	Methyl octanoate	-		-		10.17	(15)	5.98	(2)	-		-		0.93	(13)	1.76	(15)	-		-	
68	33.66	1398	1391	Butyl methylbutanoate	-		-		21.27	(12)	7.13	(1)	2.61	(3)	1.33	(2)	21.82	(4)	9.65	(11)	-		-	
69	33.99	1404	1394	Ethyl 2-hydroxyisovalerate	-		-		36.49	(10)	10.24	(2)	2.66	(17)	2.25	(6)	29.38	(12)	7.58	(18)	7.22	(13)	3.23	(6)
71	35.22	1425	1436	Ethyl octanoate ^4^	3.97	(4)	2.61	(4)	4.11	(7)	4.39	(14)	72.42	(12)	42.25	(4)	16.89	(16)	16.83	(17)	47.25	(4)	53.46	(11)
78	37.64	1465	1441	Isopentyl hexanoate	-		-		-		-		-		18.74	(2)	-		-		-		6.28	(9)
79	37.81	1468	1483	Ethyl 3-hydroxybutanoate ^4^	-		-		181.49	(8)	67.28	(15)	220.28	(7)	154.85	(5)	46.42	(9)	20.54	(16)	7.41	(15)	1.67	(16)
89	41.82	1532	1533	Hexyl butanoate	-		-		12.44	(14)	7.26	(4)	-		-		-		-		-		-	
100	45.96	1597	1591	Methyl decanoate	-		0.38	(4)	6.96	(10)	25.22	(5)	-		-		8.15	(18)	5.16	(10)	17.67	(8)	1.84	(15)
104	47.59	1624	1610	Butyl octanoate	-		0.54	(5)	-		7.06	(18)	-		-		-		0.98	(7)	-		9.51	(14)
108	48.69	1643	1636	Ethyl decanoate ^4^	-		-		102.43	(12)	84.04	(8)	-		-		60.17	(19)	6.74	(15)	6.79	(12)	19.02	(13)
110	49.08	1650	1648	Ethyl benzoate ^4^	2.14	(3)	1.99	(2)	-		1.68	(2)	-		-		-		2.04	(1)	2.39	(7)	0.97	(1)
112	49.54	1657	1664	Ethyl 3-hydroxyhexanoate ^4^	-		-		5.45	(17)	40.42	(5)	6.28	(4)	27.82	(12)	1.93	(7)	58.21	(9)	5.41	(16)	7.43	(9)
116	51.36	1687	1683	Benzyl butanoate	18.43	(7)	2.27	(9)	2.01	(14)	-		-		-		-		-		17.49	(8)	2.58	(13)
117	52.22	1700	1689	Diethyl succinate ^4^	-		1.04	(14)	3.19	(14)	-		-		-		-		-		1.48	(11)	-	
120	52.69	1708	1694	Ethyl 9-decenoate	6.83	(7)	1.03	(4)	66.22	(18)	204.61	(5)	184.42	(9)	93.89	(8)	4.33	(3)	10.33	(7)	190.78	(7)	74.42	(9)
130	55.09	1747	1745	Methyl salicylate ^4^	53.55	(4)	27.22	(5)	3.86	(12)	1.97	(3)	-		4.59	(10)	-		1.59	(5)	-		0.78	(9)
134	56.42	1768	1729	3-Hexenyl hexanoate	-		-		0.68	(19)	2.06	(5)	6.97	(2)	3.53	(10)	20.76	(16)	1.23	(2)	3.25	(8)	4.63	(14)
137	57.41	1783	1803	2-Phenylethyl acetate ^4^	27.70	(7)	2.12	(11)	59.28	(13)	16.31	(7)	12.34	(17)	7.78	(8)	4.50	(8)	12.12	(2)	1.06	(2)	10.47	(8)
141	58.86	1807	1821	Benzyl propanoate	-		-		0.54	(7)	1.98	(4)	-		-		168.13	(14)	42.07	(5)	10.62^b^	(18)	10.74^b^	(4)
146	60.32	1839	1837	Ethyl dodecanoate ^4^	-		1.07	(9)	-		22.39	(18)	-		-		-		5.47	(6)	-		27.04	(12)
148	61.85	1872	1872	Geranyl butanoate	4.23	(4)	2.10	(18)	0.74	(17)	9.42	(15)	-		-		9.88	(4)	2.37	(11)	-		22.58	(5)
151	63.49	1906	1895	Geranyl isovalerate	-		1.71	(13)	0.50	(6)	-		-		-		64.89	(14)	2.89	(4)	-		-	
154	65.54	1944	1960	Geranyl valerate	-		-		-		-		-		-		-		-		1.98	(17)	-	
155	66.22	1956	1974	Methyl jasmonate	11.10	(4)	21.96	(11)	15.26	(7)	29.99	(9)	-		-		-		-		-		-	
161	73.02	2126	2114	Bornyl benzoate	-		-		-		-		7.79	(11)	-		4.47	(14)	18.77	(10)	-		-	
163	74.72	2135	2139	Ethyl cinnamate ^4^	-		2.89	(9)	2.15	(4)	6.64	(7)	1.02 ^c^	(14)	0.98 ^c^	(2)	37.80	(19)	1.45	(10)	3.40	(17)	2.97	(7)
168	77.99	2154	2171	Diethyl 2-hydroxyglutarate	1.28	(2)	0.52	(12)	0.39	(5)	0.15	(4)	-		-		4.77	(10)	2.15	(16)	2.34	(8)	2.03	(10)
				% RPA	25.98		18.45		64.16		50.29		23.58		20.90		58.84		45.15		55.00		57.83	
				**Alcohols**																				
6	7.62	952	929	Ethanol ^4^	106.00	(4)	82.28	(9)	51.19	(15)	119.72	(9)	67.62	(12)	89.99	(8)	185.09	(14)	111.82	(12)	93.32	(6)	136.76	(14)
14	11.29	1023	1037	1-Propanol ^4^	40.25	(3)	11.05	(5)	52.66	(7)	84.80	(4)	4.26	(3)	3.98	(2)	4.17	(7)	3.63	(13)	10.21	(3)	24.98	(2)
27	16.86	1142	1099	2-Butanol ^4^	2.80	(5)	0.35	(6)	-		-		18.87	(6)	21.16	(6)	11.99	(17)	6.14	(5)	24.91	(4)	1.80	(9)
37	20.56	1201	1206	3-Methyl-1-butanol ^4^	1.83	(2)	0.99	(7)	2.15	(3)	4.25	(2)	40.21	(2)	55.17	(4)	8.39	(18)	7.23	(2)	109.54	(4)	99.08	(2)
59	29.87	1344	1332	2-Heptanol ^4^	33.40	(6)	30.15	(8)	13.20	(10)	53.42	(4)	36.18	(17)	4.01	(1)	160.92	(10)	296.92	(8)	6.78	(19)	13.78	(3)
60	30.59	1355	1360	1-Hexanol ^4^	-		-		-		6.89	(5)	-		-		-		-		-		7.00	(10)
62	31.93	1374	1379	3-Ethoxypropanol	2.55	(6)	0.83	(10)	7.36	(10)	18.74	(9)	6.42	(17)	8.26	(2)	-		-		-		19.92	(2)
67	33.31	1394	1391	3-Hexen-1-ol isomer ^4^	6.95	(5)	0.89	(3)	23.25	(8)	66.75	(5)	13.55	(13)	25.46	(5)	3.37	(16)	4.28	(8)	17.11	(10)	28.55	(2)
74	36.18	1441	1445	1-Octen-3-ol ^4^	3.04	(14)	2.20	(3)	4.27	(2)	3.33	(4)	8.90	(7)	6.30	(15)	8.57	(5)	21.73	(6)	1.19	(18)	15.40	(3)
75	36.37	1444	1467	Heptanol ^4^	-		-		27.03	(3)	9.14	(8)	12.33	(18)	9.70	(3)	-		-		8.51	(6)	13.99	(15)
81	38.61	1480	1487	2-Ethyl-1-hexanol ^4^	10.47	(9)	9.44	(13)	1.56	(9)	7.55	(9)	2.82	(10)	10.25	(2)	4.43	(13)	30.86	(5)	5.58	(8)	22.00	(7)
91	42.69	1546	1553	1-Octanol ^4^	6.62	(2)	7.29	(8)	20.37	(4)	76.80	(4)	72.34	(9)	22.57	(3)	27.20	(5)	68.14	(4)	24.58	(11)	75.42	(2)
109	48.80	1645	1624	1-Nonanol	3.45	(12)	3.78	(18)	4.67	(20)	29.29	(6)	23.40	(7)	46.54	(9)	-		-		-		-	
147	60.80	1850	1865	Benzyl alcohol ^4^	1.96	(14)	3.47	(12)	1.51	(13)	30.96	(3)	-		-		9.62	(15)	5.55	(2)	-		-	
150	62.97	1896	1925	Phenylethyl alcohol ^4^	19.48	(6)	22.53	(7)	1.92	(5)	6.72	(10)	3.10	(8)	8.23	(4)	13.47	(3)	31.77	(17)	11.77	(4)	10.93	(13)
156	67.46	1978	1952	1-Tridecanol	-		0.77	(4)	-		-		-		-		-		-		-		7.71	(17)
				% RPA	18.14		19.97		7.14		15.08		10.05		9.67		16.21		20.74		10.14		15.60	
				**Carbonyl compounds**																				
2	6.46	921	-	Butanal ^4^	-		-		5.47	(18)	9.43	(17)	4.25	(2)	9.50	(3)	3.19	(5)	7.42	(16)	1.70	(15)	5.24	(11)
4	7.19	941	926	2-Methylbutanal	3.34	(2)	1.75	(1)	2.34	(7)	1.97	(2)	-		-		-		-		0.26	(11)	2.38	(9)
5	7.33	944	936	3-Methylbutanal ^4^	4.63	(4)	1.69	(14)	-		-		-		-		-		-		-		-	
9	8.98	982	973	2-Pentanone	-		-		1.08 ^d^	(2)	0.96 ^d^	(3)	7.34	(5)	2.14	(12)	30.36	(5)	2.87	(8)	6.22	(5)	0.77	(4)
20	13.31	1072	1084	Hexanal ^4^	17.26	(11)	2.06	(18)	18.35	(7)	9.78	(12)	18.53	(12)	14.47	(7)	-		-		18.57	(18)	7.93	(6)
24	15.93	1125	1131	2-Pentenal	-		2.44	(9)	-		3.02	(13)	-		-		-		-		-		1.28	(15)
32	18.95	1177	1170	2-Heptanone	-		2.12	(1)	-		1.27	(18)	-		-		-		1.08	(10)	-		3.51	(16)
33	19.13	1179	1174	Heptanal ^4^	21.96	(10)	24.03	(7)	5.36	(17)	12.33	(5)	-		-		-		0.34	(8)	1.66	(13)	4.05	(7)
36	20.22	1196	1192	2-Hexenal isomer	22.33	(1)	25.43	(6)	36.36	(5)	27.90	(1)	10.24	(3)	20.25	(2)	3.27	(11)	38.82	(4)	8.61	(16)	20.50	(13)
38	21.17	1211	1200	2-Hexenal isomer	4.66	(10)	2.89	(10)	17.01	(15)	19.74	(5)	-		-		21.20	(5)	3.08	(2)	31.28	(13)	-	
43	23.49	1248	1244	3-Octanone ^4^	14.11	(9)	1.51	(9)	0.52	(12)	5.30	(8)	-		-		0.41	(1)	-		-		1.90	(12)
46	25.58	1279	1272	3-Hydroxy-2-butanone	9.30	(9)	0.97	(4)	-		-		-		-		-		13.09	(10)	-		1.18	(17)
47	25.73	1281	1280	Octanal ^4^	-		7.10	(6)	8.63	(3)	3.73	(10)	99.19	(13)	122.34	(4)	-		-		-		18.05	(5)
48	26.58	1292	1287	1-Octen-3-one ^4^	-		18.29	(7)	-		-		-		-		-		-		-		4.89	(9)
53	27.92	1313	1299	2-Heptenal isomer	-		-		6.75	(3)	4.23	(6)	4.53	(16)	10.89	(9)	1.57	(15)	4.26	(3)	6.51	(4)	1.02	(6)
55	28.90	1329	1319	6-Methyl-5-hepten-2-one	25.18	(7)	3.10	(15)	-		-		3.57	(3)	8.24	(11)	24.52	(10)	12.91	(6)	119.93	(11)	40.24	(8)
63	32.20	1378	1388	2-Nonanone	2.24	(1)	16.47	(8)	-		-		4.74	(1)	1.91	(7)	0.98	(2)	1.63	(9)	2.22	(10)	2.83	(1)
65	32.51	1383	1385	Nonanal ^4^	19.70	(15)	0.91	(13)	72.47	(9)	18.60	(13)	46.59	(7)	41.59	(13)	15.56	(1)	14.10	(3)	10.13	(13)	17.48	(10)
66	33.17	1392	1401	2,4-Heptadienal isomer	-		-		10.93	(1)	7.66	(4)	2.25	(1)	2.86	(6)	-		-		1.20	(4)	1.94	(5)
70	34.73	1417	1408	2-Octenal isomer	7.59	(15)	0.43	(5)	1.32	(14)	0.96	(6)	3.58	(19)	5.86	(2)	18.06	(7)	-		35.95	(8)	14.73	(9)
82	38.78	1483	1458	Methional ^4^	1.41	(3)	2.42	(8)	3.53	(5)	6.54	(14)	10.12	(14)	15.97	(3)	1.13	(18)	13.33	(7)	0.67	(7)	2.14	(2)
83	39.12	1488	1484	Decanal ^4^	40.95	(6)	14.28	(15)	37.57	(13)	16.69	(12)	84.32	(7)	35.24	(1)	15.61	(3)	11.92	(15)	14.23	(18)	28.73	(8)
86	40.58	1512	1495	Benzaldehyde ^4^	25.94	(12)	21.09	(8)	5.38	(17)	18.16	(2)	2.67	(18)	14.01	(12)	16.05	(8)	3.19	(3)	6.91	(11)	24.45	(2)
87	41.30	1524	1527	2-Nonenal isomer ^4^	-		-		6.10	(9)	9.37	(14)	10.19	(11)	16.22	(2)	5.74	(13)	1.59	(8)	3.01	(12)	9.74	(19)
95	44.40	1573	1575	2,6-Nonadienal isomer	1.46	(1)	6.51	(5)	1.08 ^e^	(15)	0.98 ^e^	(1)	-		-		-		2.07	(16)	-		-	
96	44.77	1579	1561	6-Methyl-3,5-heptadiene-2-one	-		2.47	(12)	12.57	(19)	6.44	(2)	-		-		-		3.18	(15)	-		4.35	(9)
106	48.08	1633	1645	Acetophenone ^4^	9.26	(1)	1.05	(17)	9.61	(5)	7.82	(9)	-		-		17.96	(18)	3.14	(2)	-		-	
127	54.61	1739	1710	2,4-Decadienal ^4^	-		3.17	(14)	1.66	(11)	6.89	(17)	-		-		10.06	(6)	9.62	(15)	-		34.88	(9)
129	54.96	1745	1753	Ethyl benzaldehyde ^4^	-		-		-		-		-		10.48	(4)	-		2.75	(9)	-		-	
136	57.07	1778	1758	2,4-Decadienal	-		0.23	(5)	-		-		11.22	(6)	7.15	(2)	5.39	(11)	3.27	(3)	8.91	(15)	2.37	(4)
164	75.14	2138	2103	γ-Decalactone ^4^	-		1.07	(5)	-		10.56	(9)	-		-		-		1.36	(11)	-		-	
166	76.38	2145	2112	Hexadecanone	-		0.61	(12)	0.60	(10)	2.75	(11)	-		-		-		-		-		-	
169	80.45	2167	2153	Palmitaldehyde	-		-		25.00	(12)	17.37	(14)	-		-		-		-		-		-	
				% RPA	17.57		18.62		9.80		6.70		10.48		10.53		7.08		5.47		8.99		8.39	
				**Terpenoids**																				
12	10.45	1000	1032	α-Pinene ^4^	-		-		-		-		79.43	(4)	38.60	(5)	-		5.59	(16)	6.55	(18)	1.47	(6)
17	12.26	1047	1075	Camphene	-		-		-		-		1.17	(13)	0.98	(6)	-		1.03	(3)	-		-	
21	14.03	1087	1116	β-Pinene ^4^	-		-		-		-		4.84	(3)	-		-		6.22	(14)	-		5.23	(11)
23	15.90	1124	1148	δ-3-Carene	-		-		-		-		9.57	(11)	8.96	(5)	-		-		-		-	
29	17.73	1157	1141	β-Myrcene ^4^	-		-		0.72	(7)	7.17	(5)	16.13	(12)	4.96	(2)	-		-		-		-	
35	19.68	1188	1198	Limonene ^4^	5.61	(5)	1.16	(6)	0.96	(4)	7.72	(10)	359.17	(9)	203.95	(3)	14.12	(3)	1.81	(6)	9.31	(4)	11.47	(6)
39	21.92	1224	1225	Ocimene	-		-		-		-		55.58	(3)	99.12	(3)	8.89	(14)	12.83	(7)	-		12.73	(12)
42	22.85	1239	1238	γ-Terpinene	20.74	(7)	1.75	(8)	-		-		20.58	(16)	8.16	(4)	-		-		-		-	
45	25.06	1271	1261	*p*-Cymene ^4^	-		-		-		-		76.29	(9)	99.40	(11)	-		-		-		-	
58	29.73	1342	1337	Rose oxide isomer	-		-		1.05	(5)	1.36	(12)	-		-		-		-		-		-	
72	35.47	1429	1423	Linalool oxide isomer	-		-		2.98	(2)	3.17	(15)	15.28	(7)	12.36	(3)	2.14	(5)	24.11	(11)	10.13	(2)	5.48	(1)
73	35.58	1431	1449	Dihydrolinalool	83.01	(5)	48.23	(8)	-		-		-		-		-		-		-		-	
80	37.90	1469	1488	Citronellal ^4^	6.33	(2)	4.23	(5)	-		-		26.50	(15)	12.15	(6)	-		-		0.95	(17)	1.20	(8)
84	39.77	1498	1491	Camphor	17.77	(15)	2.93	(10)	3.26	(10)	3.21	(11)	7.98	(5)	8.22	(4)	8.05	(14)	25.32	(6)	1.79	(7)	7.35	(11)
85	40.32	1507	1506	Isoborneol	7.60	(9)	6.11	(2)	-		-		-		-		24.88	(15)	7.13	(2)	26.74	(7)	11.44	(8)
88	41.61	1529	1516	Isocitral	16.34	(15)	1.63	(6)	10.86	(15)	5.51	(3)	-		-		3.52	(3)	5.51	(10)	0.96	(13)	3.48	(13)
90	42.18	1538	1537	Linalool ^4^	60.66	(2)	18.25	(4)	254.57	(17)	375.08	(4)	46.40	(17)	198.03	(4)	5.54	(9)	24.57	(8)	7.48	(4)	41.07	(6)
92	43.37	1557	1550	Dihydrocarvone	-		-		-		-		40.21	(3)	58.42	(5)	2.76	(2)	4.43	(9)	1.32	(13)	3.73	(11)
94	44.12	1569	1574	Fenchyl alcohol	-		-		4.67	(12)	4.88	(10)	64.49	(6)	24.65	(6)	20.45	(18)	3.72	(11)	15.76	(4)	30.35	(5)
97	45.21	1585	1580	Bornyl acetate	-		-		3.18	(13)	37.19	(1)	12.33	(12)	16.01	(6)	10.25	(3)	14.70	(3)	1.95	(15)	7.62	(10)
98	45.58	1591	1562	Isodihydrocarveol	-		-		-		-		25.67	(6)	65.65	(5)	0.78	(4)	1.84	(7)	1.64	(13)	9.09	(5)
99	45.89	1596	1596	Mrytenal	39.62	(6)	6.52	(3)	-		-		12.08	(6)	11.93	(14)	-		-		-		-	
101	46.15	1599	1598	β-Ciclocitral ^4^	13.87	(7)	1.25	(2)	24.00	(12)	6.40	(4)	1.26	(9)	16.60	(8)	32.96	(4)	11.92	(7)	62.18	(11)	9.01	(7)
102	46.53	1606	1602	Citral ^4^	11.21	(14)	5.50	(6)	2.98	(2)	3.17	(5)	5.01	(13)	7.72	(6)	4.96	(3)	5.21	(3)	1.53	(1)	2.96	(2)
103	46.99	1614	1646	β-Terpineol ^4^	53.04	(11)	20.97	(4)	34.86	(15)	26.29	(7)	6.56	(8)	14.37	(10)	10.01	(3)	2.70	(8)	1.21	(2)	4.88	(7)
105	47.70	1626	1648	Safranal	-		-		-		-		49.88	(8)	55.30	(12)	-		-		-		-	
107	48.44	1639	1639	Carvone	-		-		-		-		-		-		4.21	(2)	4.02	(5)	9.15	(8)	2.16	(18)
111	49.37	1654	1655	Estragole	-		-		8.27	(15)	4.27	(10)	-		-		41.13	(16)	32.61	(9)	31.53	(14)	13.11	(9)
113	50.21	1668	1648	β-Farnesene isomer	-		-		14.23	(5)	8.96	(3)	54.22	(2)	90.62	(8)	5.01	(3)	16.44	(12)	1.57	(17)	2.33	(12)
114	50.80	1678	1688	α-Terpineol ^4^	21.09	(6)	13.47	(9)	9.70	(1)	12.44	(9)	40.86	(7)	29.87	(7)	15.12	(4)	11.91	(11)	10.18	(7)	15.52	(1)
115	51.03	1681	1667	Neral	-		-		-		-		-		-		4.06	(8)	2.06	(3)	5.59	(5)	1.34	(8)
118	52.30	1701	1705	Germacrene D	-		-		2.76	(9)	1.96	(5)	128.16	(4)	18.84	(5)	1.77	(17)	1.66	(12)	9.10	(8)	11.36	(4)
119	52.57	1706	1715	Geranial isomer	-		-		-		-		-		-		34.53	(11)	19.82	(5)	-		-	
121	53.05	1714	1718	Naphthalene	4.28	(1)	8.10	(13)	-		-		-		-		3.00	(6)	3.80	(14)	17.96	(2)	19.54	(5)
122	53.26	1717	1716	TDN	12.46	(6)	1.79	(4)	-		-		3.16	(11)	-		-		-		-		0.99	(9)
123	53.65	1724	1726	Valencene	-		-		-		-		24.14	(7)	-		11.69	(7)	-		9.51	(10)	-	
124	53.91	1728	1725	α-Farnesene	-		-		13.75	(19)	20.96	(5)	27.25	(4)	21.86	(7)	18.58	(10)	27.96	(12)	419.32	(3)	8.16	(5)
125	54.10	1731	1714	α-Muurolene	-		-		-		-		38.90	(1)	31.01	(8)	12.77	(9)	1.80	(1)	-		-	
126	54.27	1734	1711	Geranyl acetate	-		-		-		-		31.54	(4)	66.51	(10)	-		-		23.65	(12)	13.37	(13)
128	54.74	1741	1715	Geranial isomer	14.43	(13)	0.50	(3)	-		-		50.82	(13)	72.06	(10)	0.98	(2)	1.97	(2)	3.61	(5)	5.64	(12)
131	55.43	1752	1751	Carvone	-		-		-		-		66.83	(3)	103.53	(8)	-		-		-		-	
132	55.82	1758	1759	Cumin aldehyde	-		-		3.88	(4)	9.76	(7)	2.48	(16)	15.89	(12)	-		-		-		-	
133	55.95	1760	1762	β-Citronellol ^4^	-		-		10.91	(16)	8.67	(10)	-		-		7.43	(5)	34.98	(5)	23.08	(15)	1.84	(11)
135	56.53	1769	1770	Nerol	7.34	(4)	4.67	(7)	-		-		82.71	(11)	87.95	(9)	-		-		2.87	(1)	1.59	(3)
138	57.50	1784	1765	Perillaldehyde	17.32	(10)	10.26	(9)	8.62	(4)	12.92	(5)	1.36	(3)	2.15	(4)	1.83	(9)	45.48	(10)	19.86	(6)	22.03	(10)
139	58.01	1792	1813	β-Damascenone ^4^	6.98	(3)	10.03	(13)	2.41	(14)	20.16	(2)	1.34	(9)	1.21	(2)	5.65	(11)	4.37	(4)	1.10	(8)	0.92	(5)
140	58.38	1798	1786	Cadinadiene	-		-		1.74	(15)	2.76	(1)	16.07	(18)	33.65	(12)	22.32	(17)	17.16	(6)	-		-	
142	59.08	1812	1809	α-Ionone ^4^	-		0.53	(6)	-		8.66	(6)	-		-		-		-		-		10.22	(9)
144	59.60	1824	1840	Geranyl acetone isomer	1.76	(1)	3.27	(4)	-		-		109.91	(15)	73.38	(7)	7.53	(1)	6.58	(6)	-		27.62	(15)
145	60.10	1834	1839	trans-carveol	-		2.29	(7)	-		-		8.23	(16)	11.22	(9)	9.89	(7)	4.08	(9)	-		-	
149	62.13	1878	1847	Geraniol ^4^	-		-		9.17	(1)	11.90	(10)	-		-		16.53	(8)	6.74	(14)	-		-	
152	64.72	1929	1912	β-Ionone ^4^	-		1.19	(16)	1.01	(8)	15.77	(7)	8.42	(15)	-		29.36	(16)	84.02	(2)	1.27	(14)	-	
153	64.98	1933	1927	Calamenene	-		-		-		-		-		-		4.86	(4)	3.73	(4)	2.14	(16)	1.98	(2)
157	68.40	1994	2009	Nerolidol ^4^	3.09	(6)	2.47	(14)	-		-		-		-		2.81	(12)	1.96	(3)	-		-	
158	69.19	2103	2089	Elemol	-		-		0.61	(2)	0.87	(2)	3.05	(11)	22.75	(12)	18.99	(7)	12.15	(3)	-		-	
159	71.74	2118	2113	Cedrenol	4.21	(3)	6.03	(4)	91.13	(11)	75.19	(11)	-		-		3.69	(2)	35.73	(6)	2.73	(8)	1.19	(3)
162	73.21	2127	2129	Spathulenol	3.98	(1)	5.33	(7)	0.73	(1)	20.72	(7)	-		-		19.20	(14)	1.13	(13)	-		-	
				% RPA	32.87		21.38		17.69		20.86		53.03		51.16		16.77		19.07		24.06		10.77	
				**Volatile phenols**																				
165	75.82	2142	2141	Eugenol ^4^	7.97	(7)	1.12	(13)	5.01	(5)	6.20	(8)	10.10	(1)	2.98	(1)	16.87	(9)	2.65	(12)	8.54	(4)	9.63	(10)
167	76.56	2146	2170	4-Ethyl phenol ^4^	-		1.88	(14)	-		1.21	(2)	-		15.45	(16)	-		-		-		1.16	(12)
				% RPA	0.61		0.34		0.17		0.22		0.33		0.57		0.63		0.09		0.28		0.35	
				**Fatty acids**																				
76	36.53	1447	1450	Acetic acid ^4^	34.60	(5)	20.77	(6)	1.37	(15)	7.08	(8)	2.45	(12)	32.50	(5)	7.74	(11)	13.49	(8)	24.06	(3)	12.43	(6)
143	59.21	1815	1820	Hexanoic acid ^4^	9.88	(15)	2.40	(16)	26.48	(13)	18.33	(9)	68.43	(13)	58.96	(2)	1.26	(5)	2.98	(3)	8.16	(11)	1.03	(13)
160	72.60	2123	2083	Octanoic acid ^4^	15.88	(18)	2.65	(8)	2.14	(4)	12.49	(13)	2.73	(14)	14.74	(8)	2.60	(10)	2.07	(10)	11.71	(4)	5.36	(7)
				% RPA	4.58		2.93		1.01		1.10		2.39		3.30		0.43		0.65		1.42		0.62	
				**Furanic compounds**																				
77	37.12	1457	1455	2-Furfural ^4^	3.39	(1)	159.04	(4)	0.79	(7)	194.85	(7)	4.67	(6)	124.55	(10)	1.25	(1)	247.37	(4)	3.26	(5)	197.05	(4)
93	43.71	1562	1560	5-Methyl-2-furfural ^4^	-		2.40	(6)	-		2.68	(6)	-		-		-		2.73	(13)	-		-	
				% RPA	0.26		18.32		0.03		5.75		0.15		3.87		0.05		8.82		0.11		6.44	

^1^ RT: Retention time (min). ^2^ Kovat index relative n-alkanes (C_8_ to C_20_) on a BP-20 capillary column. ^3^ Kovat index relative, reported in literature for equivalent capillary column [34,41,42,43]. ^4^ Volatile organic compounds (VOCs) identified by standard; -: Not detected; TDN: 1,2-dihydro-1,1,6-trimethylnaphtalene; Different superscript lowercase letters in a row indicate no significant differences between mean values at *p* < 0.05, obtained by one-way ANOVA, followed by Tukey’s test, for post-hoc multiple comparisons of means, for fresh and processed fruit juice (*n* = 2).

## Supplementary Materials

The following are available online at https://www.mdpi.com/1420-3049/24/5/974/s1, Figure S1: Total ion chromatograms obtained by HS-SPME_DVB/CAR/PDMS_/GC-qMS analysis of processed fruit juices (for identification of peak numbers see Table 1), Figure S2: Overload of GC-qMS chromatograms (enlarged part of the chromatograms of Figure 1) showing the comparison of the typical profile of 2-furfural identified in fresh and processed fruit juices, Figure S3: Hierarchical cluster analysis (HCA). The heat maps of the 169 VOCs identified in fresh and processed fruit juices were generated by average algorithm and Pearson distance analysis (attribution of the peak number is shown in Table 1). Abbreviation: OP—orange juice processed, OF—orange juice fresh, GP—grape juice processed, GF—grape juice fresh, PP—pear juice processed, PF—pear juice fresh, ApP—apple juice processed, ApF—apple juice fresh, Table S1: Common volatile organic compounds (VOCs) identified in fresh and processed fruit juices, Table S2: Predominant VOCs in fresh and processed fruit juices analyzed using HS-SPME/GC-MS methodology.
