# Supplementary material for: Differentiation of Fresh and Processed Fruit Juices Using Volatile Composition
Source: Molecules. 2019 Mar 10;24(5):974. doi: 10.3390/molecules24050974 (PMC6429179; doi:10.3390/molecules24050974)
Supplement: Supplementary file 1 [file molecules-24-00974-s001.pdf]

Supplementary Material

Differentiation of Fresh and Processed Fruit Juices using Volatile Composition

Rosa Perestrelo [1,*], Catarina Silva [1], Pedro Silva [1], Sonia Medina [1] and José S. Câmara [1,2]

[1] CQM–Centro de Química da Madeira, Universidade da Madeira, Campus da Penteada, 9020-105 Funchal, Portugal; rmp@uma.pt, cgsluis@uma.pt, pedro_dasilva@hotmail.com, sonia.escudero@staff.uma.pt

[2] Departamento de Química, Faculdade de Ciências Exatas e Engenharia, Universidade da Madeira; Campus da Penteada, 9020-105 Funchal, Portugal; jsc@staff.uma.pt

* Correspondence: rmp@uma.pt; Tel.: +351 291705119

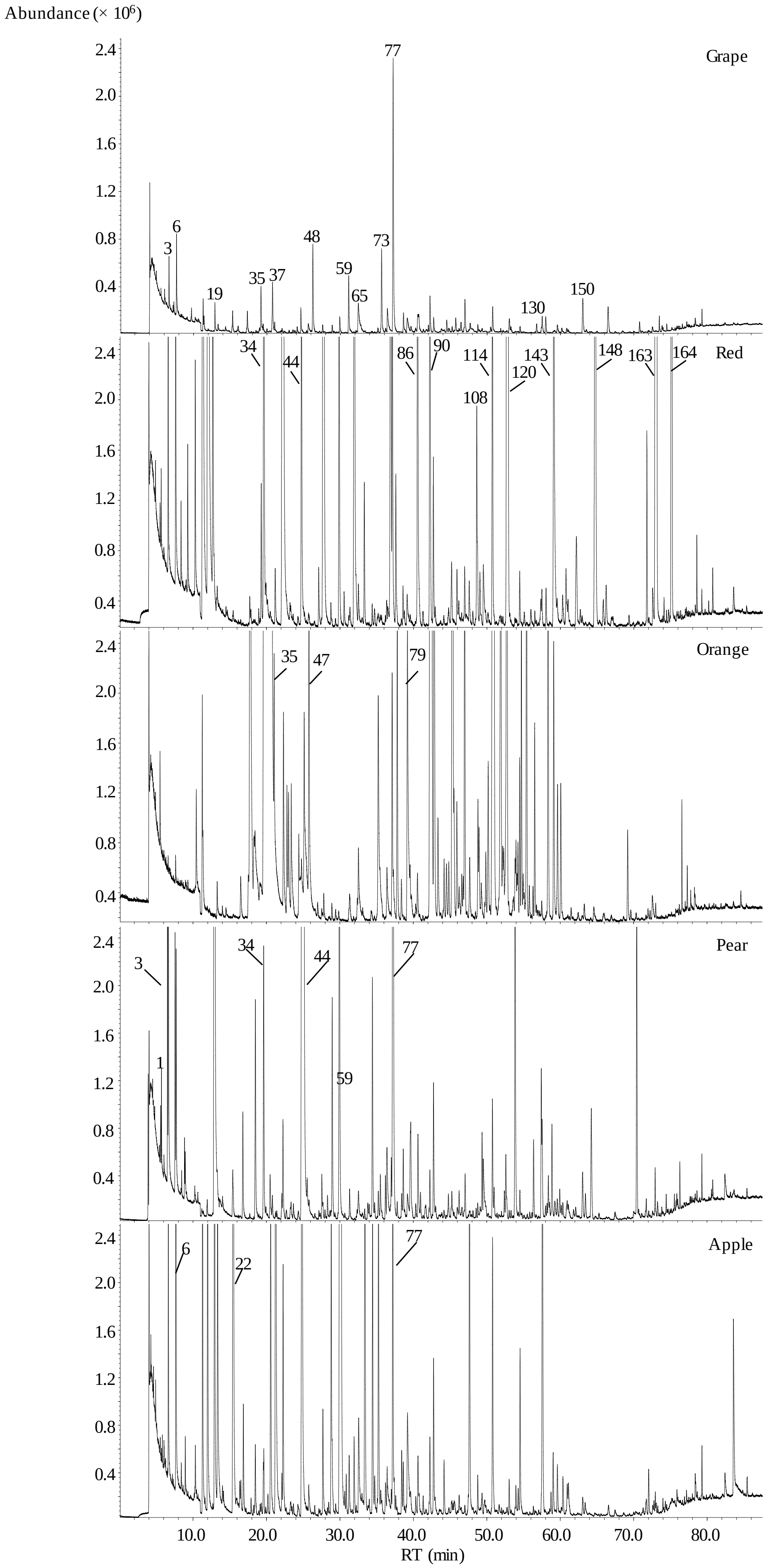

Abundance (× 10^6)
Grape
Red
Orange
Pear
Apple
2.4
2.0
1.6
1.2
0.8
0.4
3
6
19
35
37
48
59
65
73
77
130
150
34
44
86
90
108
114
120
143
148
163
164
35
47
79
3
1
34
44
59
77
6
22
77
10.0
20.0
30.0
40.0
50.0
60.0
70.0
80.0
RT (min)

Figure S1: Total ion chromatograms obtained by HS-SPMEDVB/CAR/PDMS/GC-qMS analysis of processed fruit juices (for identification of peak numbers see **Table 1**).

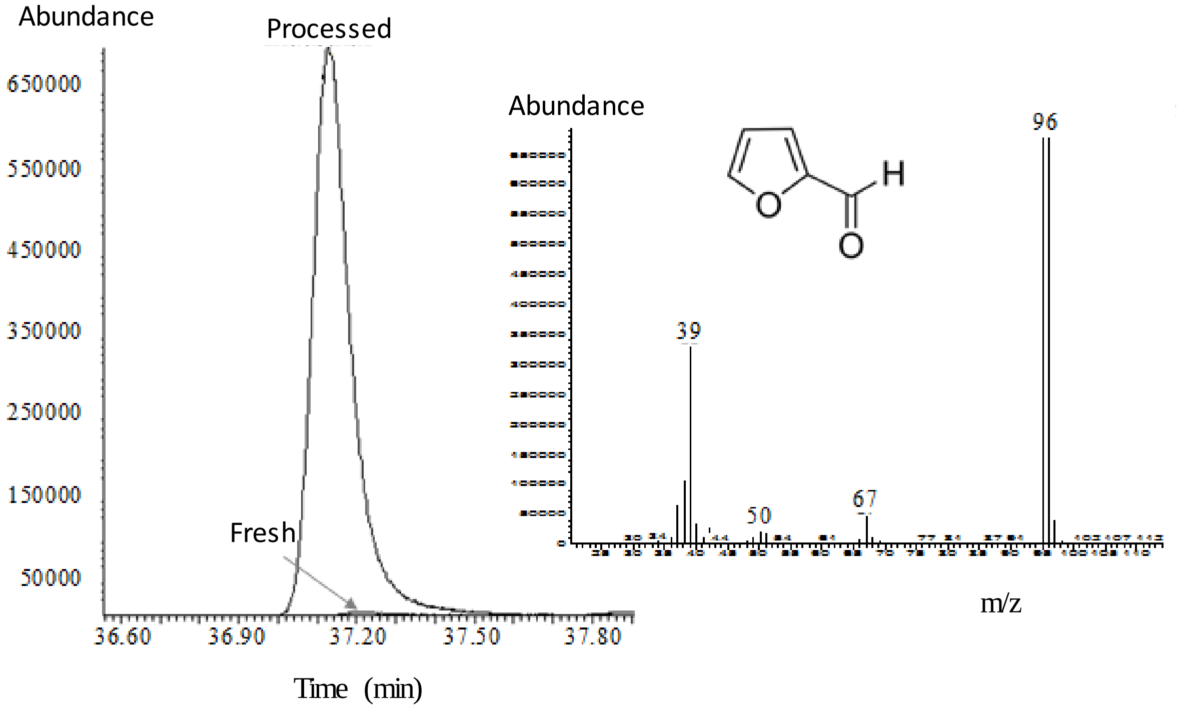

Figure S2: Overload of GC-qMS chromatograms (enlarged part of the chromatograms of Figure 1) showing the comparison of the typical profile of 2-furfural identified in fresh and processed fruit

juices.

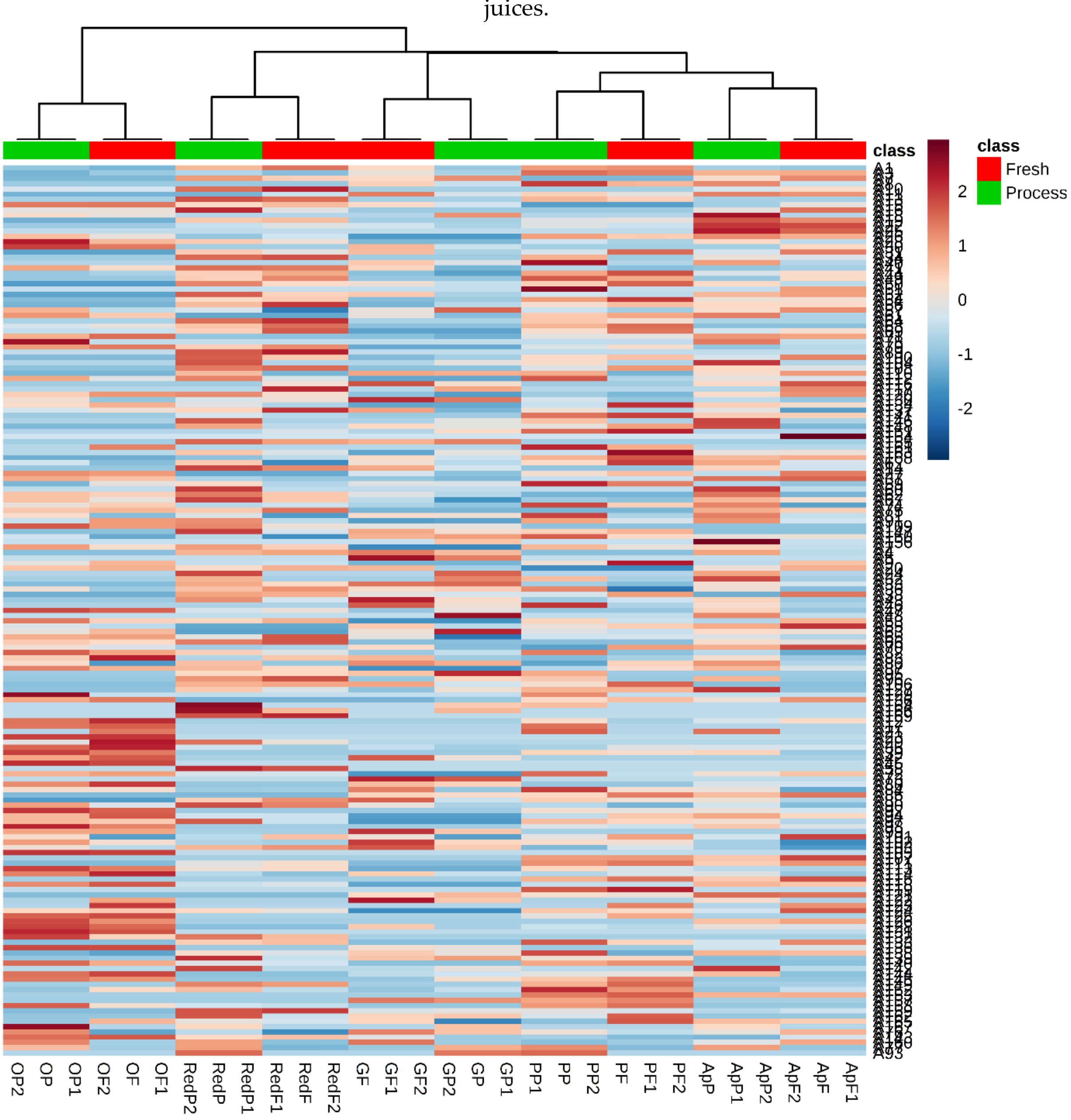

Figure S3: Hierarchical cluster analysis (HCA). The heat maps of the 169 VOCs identified in fresh and processed fruit juices were generated by average algorithm and Pearson distance analysis (attribution of the peak number is shown in Table SM1). Abbreviation: OP – orange juice processed, OF – orange juice fresh, GP – grape juice processed, GF – grape juice fresh, PP – pear juice processed, PF – pear juice fresh, ApP – apple juice processed, ApF – apple juice fresh.

Table S1: Common volatile organic compounds (VOCs) identified in fresh and processed fruit juices.

Chemical groups	VOCs
Esters	methyl acetate, ethyl acetate, ethyl hexanoate, hexyl acetate, ethyl 2-hexenoate, ethyl octanoate, ethyl 9-decenoate, 2-phenylethyl acetate
Alcohols	ethanol, 1-propanol, 3-methyl-1-butanol, 2-heptanol, 3-hexen-1-ol isomer, 1-octen-1-ol, 2-ethyl-1-hexanol, 1-octanol, phenylethyl alcohol
Carbonyl compounds	2-hexenal isomer, nonanal, methional, decanal, benzaldehyde
Terpenoids	limonene, camphor, linalool, β-ciclocitral, citral, β-terpeniol, α-terpineol, perillaldehyde, β-damascenone
Volatile phenols	eugenol
Acids	acetic acid, hexanoic acid, octanoic acid
Furanic compounds	2-furfural

Table S2: Predominant VOCs in fresh and processed fruit juices analyzed using HS-SPME/GC-MS methodology

**Juice Samples**		**VOCs predominant**
Grape	Fresh	Ethanol, dihydrolinalool, linalool, methyl salicylate, β-terpineol
	Processed	2-Furfural, ethanol, dihydrolinalool, 2-heptanol, methyl salicylate
Red	Fresh	Methyl hexanoate, hexyl acetate, linalool, ethyl 3-hydroxybutanoate, Ethyl decanoate,
	Processed	Methyl hexanoate, linalool, hexyl acetate, ethyl 9-decenoate, 2-furfural
Orange	Fresh	Limonene, ethyl 3-hydroxybutanoate, ethyl 9-decenoate, germacrene D, geranyl acetone isomer
	Processed	Limonene, linalool, ethyl 3-hydroxybutanoate, 2-furfural, octanal
Pear	Fresh	Hexyl acetate, ethyl acetate, ethanol, benzyl propanoate, 2-heptanol
	Processed	Hexyl acetate, 2-heptanol, ethyl acetate, 2-furfural, methyl hexanoate
Apple	Fresh	3-Methylbutanol acetate, α-farnesene, hexyl acetate, ethyl 9-decenoate, ethyl acetate
	Processed	3-Methylbutanol acetate, butyl acetate, 2-furfural, ethanol, ethyl acetate
